# Separate Neural Networks for Gains and Losses in Intertemporal Choice

**DOI:** 10.1007/s12264-018-0267-x

**Published:** 2018-08-07

**Authors:** Yang-Yang Zhang, Lijuan Xu, Zhu-Yuan Liang, Kun Wang, Bing Hou, Yuan Zhou, Shu Li, Tianzi Jiang

**Affiliations:** 1 School of Psychology, Shaanxi Normal University, Xi’an, 710062 China; 2 Brainnetome Center, Institute of Automation, University of Chinese Academy of Sciences, Beijing, 100190 China; 3 National Laboratory of Pattern Recognition, Institute of Automation, University of Chinese Academy of Sciences, Beijing, 100190 China; 4 CAS Key Laboratory of Behavioral Science, Institute of Psychology, Beijing, 100101 China; 5 Department of Psychology, University of Chinese Academy of Sciences, Beijing, 100049 China; 6 Department of Psychology and Behavioral Sciences, Zhejiang University, Hangzhou, 310028 China; 7 CAS Center for Excellence in Brain Science and Intelligence Technology, Chinese Academy of Sciences, Beijing, 100190 China; 8 The Clinical Hospital of Chengdu Brain Science Institute, MOE Key Lab for Neuroinformation, University of Electronic Science and Technology of China, Chengdu, 610054 China

**Keywords:** Intertemporal choice, Discounting losses, Effective connectivity, Dynamic causal model, Dorsolateral prefrontal cortex, Insula

## Abstract

An important and unresolved question is how human brain regions process information and interact with each other in intertemporal choice related to gains and losses. Using psychophysiological interaction and dynamic causal modeling analyses, we investigated the functional interactions between regions involved in the decision-making process while participants performed temporal discounting tasks in both the gains and losses domains. We found two distinct intrinsic valuation systems underlying temporal discounting in the gains and losses domains: gains were specifically evaluated in the medial regions, including the medial prefrontal and orbitofrontal cortices, and losses were evaluated in the lateral dorsolateral prefrontal cortex. In addition, immediate reward or punishment was found to modulate the functional interactions between the dorsolateral prefrontal cortex and distinct regions in both the gains and losses domains: in the gains domain, the mesolimbic regions; in the losses domain, the medial prefrontal cortex, anterior cingulate cortex, and insula. These findings suggest that intertemporal choice of gains and losses might involve distinct valuation systems, and more importantly, separate neural interactions may implement the intertemporal choices of gains and losses. These findings may provide a new biological perspective for understanding the neural mechanisms underlying intertemporal choice of gains and losses.

**Electronic supplementary material:**

The online version of this article (10.1007/s12264-018-0267-x) contains supplementary material, which is available to authorized users.

## Introduction

Decisions about daily life, such as education, diet, investment, and saving, all involve intertemporal choices, which refer to decisions between smaller/sooner and larger/later rewards or punishments [1]. People naturally devalue rewards in accordance with the delay in receiving them, which is referred to as “temporal discounting” [2–5]. Economists assume that people evaluate delayed losses in a similar way by discounting the value of outcomes according to the delay. This assumption implies that losses may recruit the same mechanisms as gains. However, several studies have reported that the aversion to losses declines more slowly than the attractiveness of gains as the delay increases, suggesting different neural correlations in intertemporal choice of gains and losses [6–12]. These findings challenge the view that gains- and losses-related temporal discounting can be condensed into a single discount function [13]. Therefore, whether a single neural process underlies the intertemporal choices in gains and losses in particular, and whether the domain of gains and losses differentially alters interactions within decision-related regions of the brain remain unclear.

Recent findings suggest that interactions between brain regions might play a critical role in intertemporal choice of gains and losses. McClure *et al.* [14, 15] originally found that temporal discounting is associated with two neural systems: the mesolimbic midbrain dopamine system is activated when making choices of immediate rewards, whereas the fronto-parietal system is activated when making choices independent of delay. Afterwards, Kable and Glimcher [16] found that a common valuation system appears to evaluate the subjective values of delayed monetary rewards, regardless of the delay time. However, Xu *et al.* found that, accompanied by the smaller/sooner choices in the losses domain than in the gains domain, different mesolimbic regions are activated in choices that involve immediate gains and losses, although lateral prefrontal regions are commonly activated in both gains and losses [17]. Consistent with this finding, a small number of studies highlighted that the nature of the interaction between these systems or regions is essential for understanding the neural mechanisms of intertemporal choice [18–20]. For example, we used the same clusters as in the Xu *et al.* study [17], and found an asymmetrical effect of approach/avoidance motivation on the functional connectivity in both the gain and loss domains [18]. However, the effective connectivity among these regions (the influence exerted by one region on another) and how the effective connectivity differed in the gains and losses domains have still not been investigated.

In the neuroscience field, two methods are commonly used to evaluate functional integration, i.e., the effective connectivity, between brain regions: psychophysiological interaction (PPI) and dynamic causal modeling (DCM). PPI identifies regions that show altered connectivity in response to the influence of a defined seed region, this connectivity being modulated by the current experimental context [21]. DCM is a nonlinear systems identification procedure that uses Bayesian estimates of parameters to make inferences about the coupling among areas and how that coupling is influenced by changes in the experimental context [22, 23]. DCM together with PPI could make inferences about the directionality of the effects and about the most likely functional architecture. Thus, these analyses would contribute to investigating whether distinct neural networks exist and how the dynamic interactions within these networks are altered between intertemporal choice in the gains and losses domains.

In the present study, using PPI and DCM analyses, we further analyzed our previous functional magnetic resonance imaging (fMRI) data to investigate the functional interactions of the brain regions involved in intertemporal choice in the gains and losses domains. We first identified the critical regions that showed activation patterns covarying with seed regions depending on experimental context using PPI analysis [21] and then inferred the causal architecture among those regions using DCM analysis [22]. Of the seed regions, we particularly focused on the ventral striatum and the medial cortical regions (including the orbitofrontal and medial prefrontal cortices), which have been implicated in reward valuation and the computation of goal values [24–29]; the dorsolateral prefrontal cortex, which exerts cognitive control of decision-making processes [29–35]; and the anterior cingulate and insula, which are involved in responding to aversive stimuli, the evaluation and representation of negative emotional states, and even pain [17, 36–41].

## Methods

### Participants

Twenty healthy, right-handed Chinese graduate students [10 females; 25.0 ± 1.7 years old (range, 22–29 years, no gender differences in age (*P* = 0.74) or educational level (*P* = 0.12)] were recruited from the Institute of Automation, University of Chinese Academy of Sciences, Beijing. All the participants had normal or corrected-to-normal vision and no history of neurological or psychiatric disorders. This study was approved by the Institutional Review Board of the Beijing MRI Center for Brain Research, and all participants provided written informed consent. Two participants, however, were excluded because of excessive head motion (absolute displacement with regard to the reference scan exceeded 2 mm).

### Experimental Task

A temporal discounting task that was previously employed by McClure *et al.* (2004) was used in our study. Specifically, we revised the original task by using a symmetric pattern of gains and losses domains, which allowed for the extraction of BOLD signal patterns associated with each single trial, and a direct comparison was then performed between gain- and loss-related brain activities. The temporal discounting task included two parts: a temporal discounting task involving gains (G-TD) and a temporal discounting task involving losses (L-TD). In both parts, the participants were asked to choose from immediate and delayed options that varied across trials. In each trial, the participants were simultaneously presented with a smaller/sooner (SS) option (e.g., “¥40 today”) and a larger/later (LL) option (e.g., “¥60 in one month”). The SS option was available at different times [selected from the set (today, two weeks from now, and one month from now)]. The delay between the LL options was either two weeks or one month. The amount of the monetary reward in the SS options ranged from ¥13 to ¥110 and was randomly drawn from a Gaussian distribution with a mean of ¥50 and a standard deviation of ¥25, with integer conversion. The percentage difference in amounts between the two rewards was selected from the set {5%, 10%, 15%, 25%, 35%, and 50%}. Participants were asked to select one option by pressing a button that corresponded to the location of the chosen option on the screen. Both tasks had identical form, with the exception that a minus sign before a monetary amount indicated that the money would be lost in the L-TD.

**Fig. 1:**
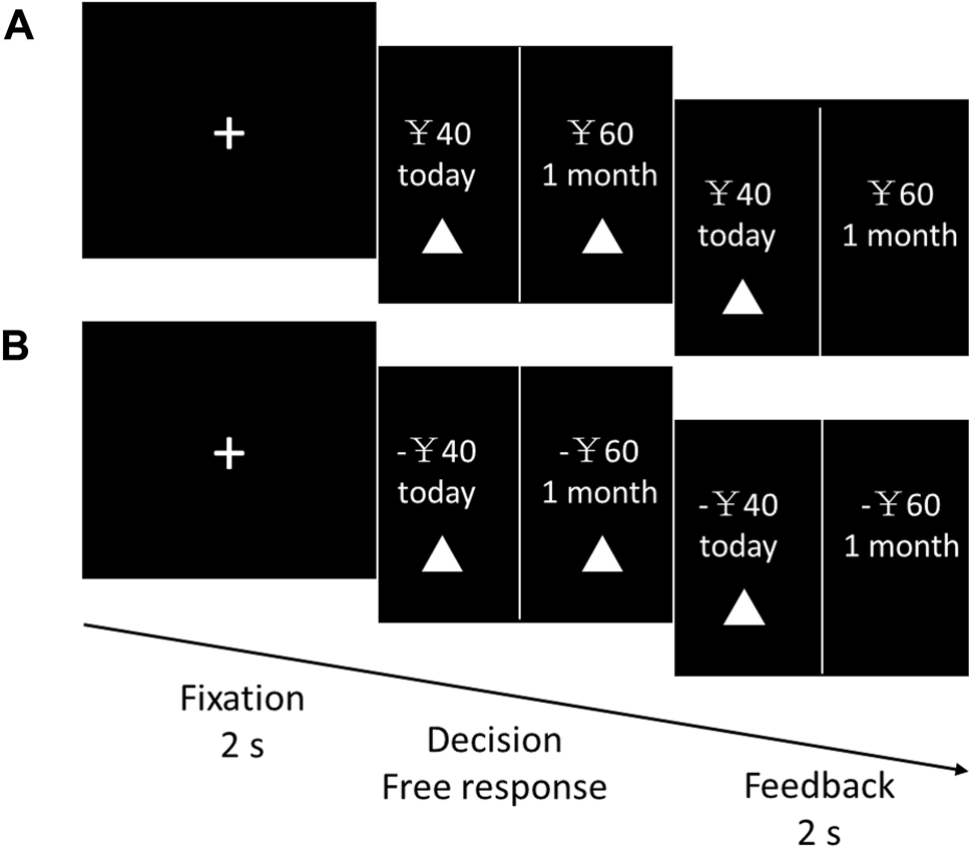
Illustration of a trial used in the experiment. **A** Trial structure for a temporal discounting task involving gain (G-TD). **B** Trial structure for a temporal discounting task involving loss (L-TD).

The procedure of the temporal discounting task is shown in Fig. 1 (also in our previous study [17]). In each trial, a 2-s fixation period was presented, which indicated the next trial. The selected result was visible for 2 s after the participant’s response, followed by a black screen for 10 s. In the instructions about the L-TD, an initial offer of ¥150, which corresponded to the maximum amount a participant could lose, was made available for each participant. Participants received the corresponding payment at the specified time in one trial which was randomly selected from each task to ensure incentive compatibility.

Besides the temporal discounting task, we also assessed the participants’ approach and avoidance personality in a separate session after the scanning, using two subscales of Zuckerman-Kuhlman Personality Questionnaire [42, 43]: the Impulsive and the Neuroticism-Anxiety subscales. As reported in our previous work [18], the mean scores of approach personality and avoidance personality of all 18 participants were 2.33 ± 1.46 and 6.67 ± 3.68, respectively. No gender differences were found for approach (*P* = 0.76) and avoidance personality (*P* = 0.26).

### fMRI Data Acquisition and Preprocessing

Imaging was performed using a 3.0T Siemens MR scanner (Erlangen, Germany) at the Beijing MRI Center for Brain Research. Functional data were acquired in 26 axial slices using an echo-planar imaging (EPI) sequence (TR/TE = 2000/30 ms, flip angle = 90°, field of view = 19.2 cm, matrix = 64 × 64, thickness = 3 mm, gap = 1 mm). High-resolution T1-weighted anatomical images were collected in the two functional runs. The first run was the G-TD, and the second was the L-TD.

The analysis was conducted using SPM8 software (Wellcome Department of Cognitive Neurology, London, UK). The first five images were discarded from the analysis to allow for magnetic saturation effects. Functional images were corrected for differences in slice acquisition timing, corrected for head motion, normalized to a standard EPI template, and then spatially smoothed with a Gaussian kernel of 8 mm full-width-at-half-maximum.

### Whole-Brain Analysis

The whole-brain analysis was performed as in our previous work [17]. Our aim was to identify regions activated during G-TD and L-TD. The whole-brain analysis was first conducted at the individual level using the voxel-wise general linear model. We separately created the motion parameters and two task-related regressors for each participant: one for trials in which the early option was available immediately, the other for all trials. Then, individual T-contrasts generated for each regressor were entered into a second-level group analysis with the one-sample *t*-test. The regions activated in each task then served as the regions of interest (ROIs) for the subsequent analysis. The statistical threshold was set to *P* < 0.05 (uncorrected). For more details of the analysis and results of whole-brain analysis, refer to Xu *et al.* [17].

### Psychophysiological Interaction Analysis

After identifying the regions involved in both G-TD and L-TD in the whole-brain analysis, we used PPI analysis to capture the interactions between these regions in relation to the experimental conditions [21]. PPI analysis allows the detection of regionally specific responses in one brain region in terms of the interaction between input from another region and a cognitive/sensory process [21]. At this stage of analysis, we used an *a priori*, hypothesis-driven method similar to those used in other areas of research [44–46].

For each participant, the regions showing a selective increase in activation in response to choices that involved immediate options in the whole-brain analysis were selected as seed regions for the PPI analysis. The selected seed regions for each individual were the medial orbitofrontal cortex (MOFC), medial prefrontal cortex (MPFC), posterior cingulate cortex (PCC), and the ventral striatum in G-TD, and the PCC, MPFC, anterior cingulate cortex (ACC), and insula in L-TD.

To perform PPI analyses, the first Eigen variate time series from a sphere of 12-mm radius centered on the most significant voxel from the seed regions were extracted. For each seed region, the effect of the interaction term was computed as the element-by-element product of the seed region time series and a vector coding the contrast of interest (1 for the immediate condition and –1 for the delayed condition). The individual contrast images were then entered into the second level to perform a random effects analysis (using a one-sample *t* test). A stringent random effects model with *a priori* defined regions and a statistical threshold of *P* < 0.005 (uncorrected) was used. For inference purposes, the regions reported in supplementary Tables S1 and S2 are corrected for multiple comparisons based on Monte Carlo simulation using the AFNI AlphaSim program [47]. A combined threshold of 0.01 and a cluster size >74 resampled voxels determined by Monte Carlo simulation were used to correct for multiple comparisons at a statistical threshold of *P* < 0.05.

The PPI analysis was an important first step in exploring the functional interactions of the activated regions in the TD task. Based on the regions detected in the PPI analysis, we used DCM analysis to further explore the effects of the stimulus input, the causal direction, and the bilinear modulatory effect of the experimental conditions in the intertemporal choice.

### Dynamic Causal Modeling Analysis

To identify the brain regions that responded to the input from all the choices and to determine how the connection strengths were modulated by the immediate condition, we used DCM to examine the directional influence between the regions that were detected using the PPI analysis. A series of subject-specific dynamic causal models were separately constructed for the G-TD and the L-TD.

**Fig. 2:**
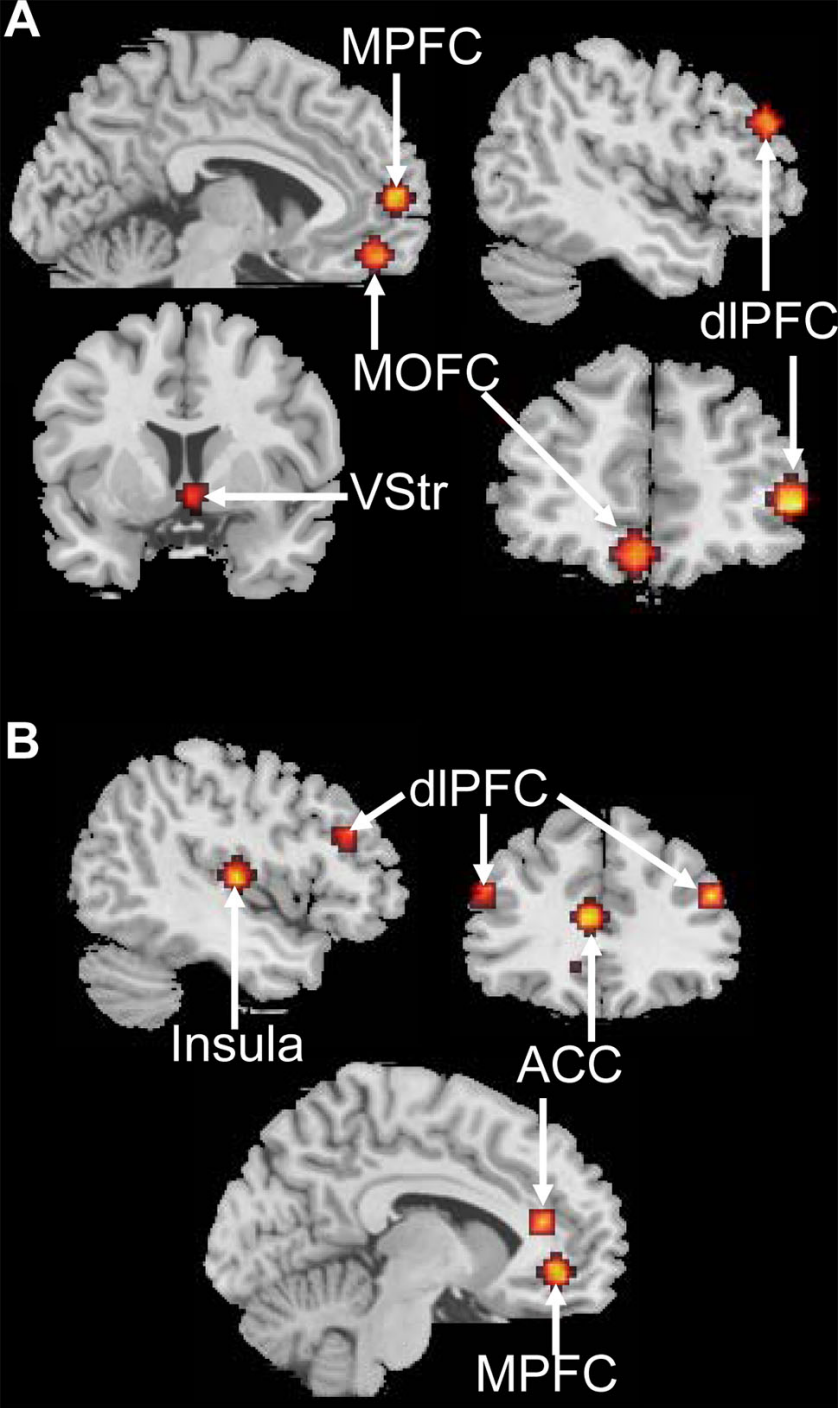
Regions of interest (ROIs) selected for DCM analysis. **A** ROIs for the G-TD. The medial orbitofrontal cortex (MOFC), medial prefrontal cortex (MPFC), and ventral striatum (VStr) were defined by conventional SPM analysis. The bilateral dorsolateral prefrontal cortices (dlPFC) were defined by their overlapping sensitivity to both the conventional and PPI analyses. **B** ROIs for the L-TD. The MPFC, anterior cingulate cortex (ACC), and insula were defined by conventional SPM analysis. The bilateral dlPFC were defined by their overlapping sensitivity to both the conventional and PPI analyses.

For the G-TD, fMRI time-courses were extracted from the MPFC, MOFC, ventral striatum, and bilateral dorsolateral prefrontal cortices (dlPFC) (Fig. 2A) that had been identified in the whole-brain and the PPI analyses. Four possible models were defined in which the stimulus inputs (i.e., the presentation of choices regardless of the delay) were connected to different parts of the network: (1) a model with stimulus inputs to all the five regions; (2) a model with stimulus inputs to the medial-striatal regions, including the MPFC, MOFC, and ventral striatum; (3) a model with stimulus inputs to the bilateral dlPFC only; and (4) a model with stimulus inputs to the medial frontal regions, including the MPFC and MOFC (Fig. S1). All the models assumed bidirectional intrinsic connections among these five regions. Because PPI analysis indicated that the interactions within the medial-striatal network and between the dlPFC and the medial-striatal network were enhanced in the immediate condition, we set the immediate condition to modulate the connections within the medial-striatal network and the connections between the dlPFC and the medial-striatal network.

For the L-TD, fMRI time-series were extracted from four regions (the MPFC, ACC, insula, and right dlPFC) (Fig. 2B) that were identified in the whole-brain and PPI analyses. The left dlPFC that came from the mirror image of the right dlPFC (48, 30, 24) was also taken into account, considering that the left dlPFC plays an essential role of cognitive control in intertemporal choice [48, 49]. Four possible models were defined in which the driving inputs were connected with different parts of the network: (1) a model with stimulus inputs to all the five regions; (2) a model with stimulus inputs to the MPFC-cingulate-insula regions; (3) a model with stimulus inputs to the bilateral dlPFC only; and (4) a model with stimulus inputs to the MPFC only (Fig. S2). All the models assumed bidirectional intrinsic connections among these five regions and assumed that the immediate condition modulated the connections within the MPFC-cingulate-insula network and the connections between the dlPFC and the MPFC-cingulate-insula network.

For each seed region in both tasks, fMRI time-series were extracted from a 12-mm sphere centered on maximum coordinates. The maximum coordinates of the MPFC, MOFC, and ventral striatum in the G-TD, and the MPFC, ACC, and insula in the L-TD were based on the whole-brain analysis, and those of the bilateral dlPFC were based on the PPI analysis (listed in Tables S3 and S4).

After constructing a series of DCMs for each participant, we compared these DCMs to separately obtain optimal models for the G-TD and the L-TD using Bayesian model selection. Once the optimal model was selected, the participant-specific immediate-related modulatory parameters were entered into group analysis with a one-sample *t* test. This allowed us to summarize the consistent findings from the subject-specific DCMs using classical statistics. Because our study was hypothesis-driven, the results at a threshold of *P* < 0.05 (uncorrected and Bonferroni corrected) are reported.

## Results

In our previous report [17], we found a significantly larger percentage of SS choices in the L-TD than in the G-TD, suggesting a significant reduction in the delay discounting for future losses compared with future gains. In addition, we found activation of the PCC, MOFC, MPFC, and ventral striatum in the G-TD as well as the PCC, MPFC, ACC, and insula in the L-TD. Having separately identified the regions that exhibited characteristic responses in G-TD and L-TD, we used effective connectivity analysis to characterize the regional integrations in intertemporal choices of gains and losses.

### G-TD

**Fig. 3:**
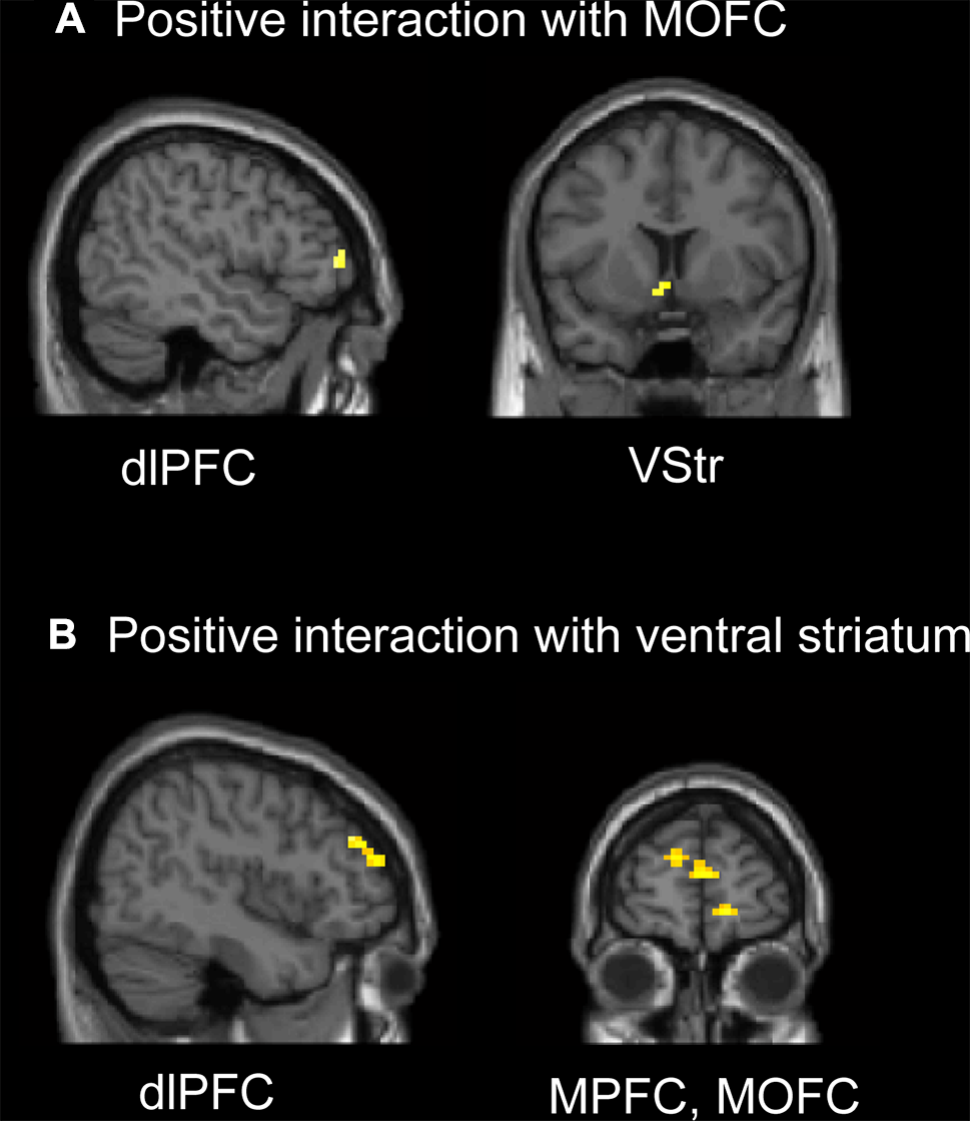
Psychophysiological interaction (PPI) results for all participants in the G-TD. **A** Regions that showed a significant interaction with activity in the medial orbitofrontal cortex (MOFC) during the immediate condition compared to the delayed condition. **B** Regions that showed a significant interaction with activity in the ventral striatum (VStr) during the immediate condition compared to the delayed condition. MPFC, medial prefrontal cortex; dlPFC, dorsolateral prefrontal cortex.

In our previous report [17], we found significant activation across all decision epochs of gains in the frontal-parietal network, including the dlPFC, PPC, and lateral orbitofrontal cortex. We also found enhanced activation in the PCC and the medial-striatal network (including the MOFC, MPFC, and ventral striatum) for choices in which money was available immediately. We focused on the lateral prefrontal cortices and the medial-striatal network in the PPI analysis because these regions have been implicated in cognitive control of the decision-making process [30–35] and subjective value evaluation [24–28]. PPI analysis revealed greater functional coupling within the medial-striatal network and between the lateral dlPFC and the medial-striatal network (including the MOFC, MPFC, and ventral striatum) when comparing the immediate condition with the delayed condition (Fig. 3, Table S1). Specifically, compared with all delayed options, the immediate options were accompanied by increased functional connectivity between the MOFC and left DLPFC (*x* = –48, *y* = 45, *z* = 3, *t*-score = 3.53), between the MPFC and right DLPFC (*x* = 45, *y* = 42, *z* = 30, *t*-score = 3.51), and between the ventral striatum and right DLPFC (*x* = 45, *y* = 42, *z* = 30, *t*-score = 4.23). The ventral striatum also showed a positive covariation with the medial frontal cortex, including the MOFC (*x* = 15, *y* = 60, *z* = -6, *t*-score = 3.71) and MPFC (*x* = 3, *y* = 60, *z* = 12, *t*-score = 4.12), when comparing the immediate condition with the delayed condition.

**Fig. 4:**
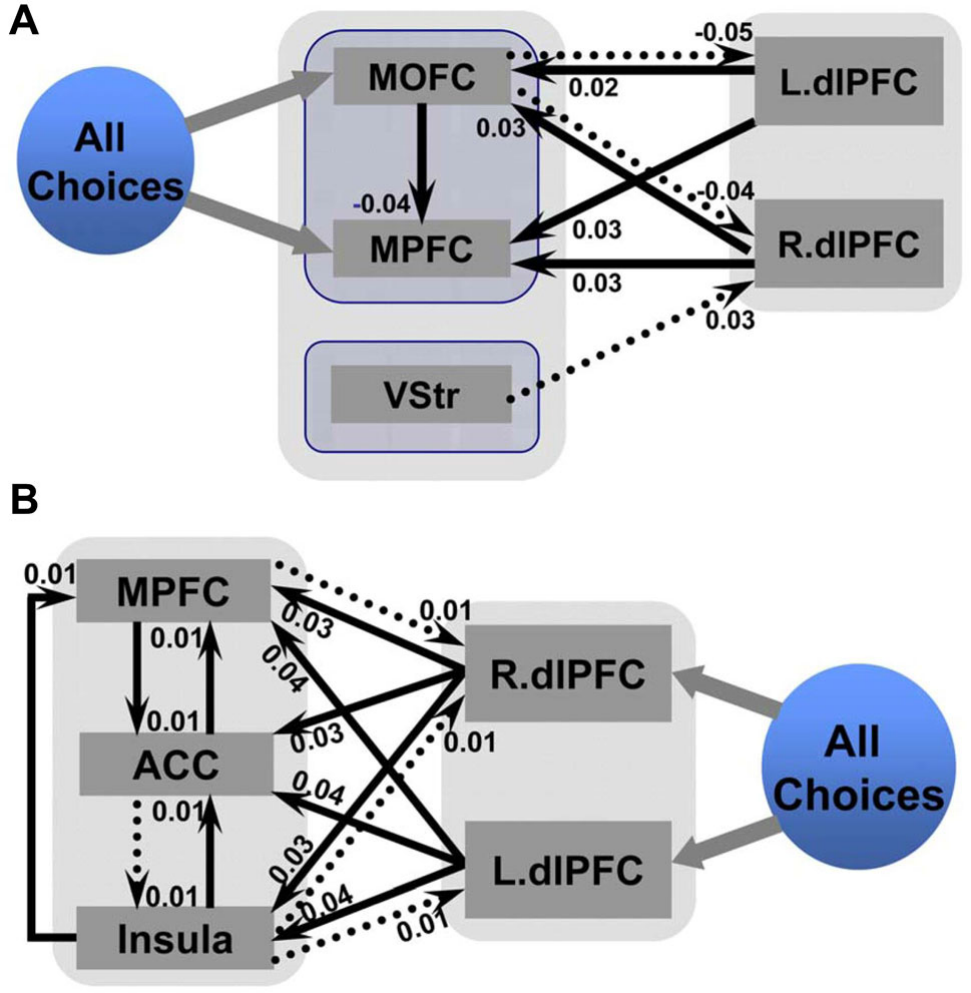
Optimal DCM in the G-TD and L-TD. Bayesian model selection indicated that the optimal DCM, chosen out of four models, was characterized by a strong impact of the experimental context (all choices, regardless of delay) on the activity of medial regions, including the MPFC and the MOFC. **A** The network in the G-TD showed significant bilinear modulatory effects in the preferred dynamic causal model that included the MPFC, MOFC, VStr, and bilateral dlPFC. **B** The network in the L-TD showed significant bilinear modulatory effects in the preferred dynamic causal model that included the MPFC, ACC, insula, and bilateral dlPFC. Values are the means of changes in connection strength induced by the immediate condition effects. These parameters quantify how experimental manipulations (immediate condition) change the values of intrinsic connections.

The DCM analysis revealed a preference for model 4 in which all choices (regardless of delay) posited a significant influence on the MPFC and MOFC (Figs. 4A and 6; also see Fig. S3). In this model, the immediate condition enhanced the connectivity from the bilateral dlPFC and the medial frontal regions, including the MPFC and MOFC. The modulating effect of the immediate condition on the connection from the ventral striatum to the dlPFC was also positive, which indicated that the effective connectivity from the ventral striatum to the dlPFC was even stronger. It should be noted that the immediate condition led to a significant “inhibitory” modulation in the activation of the pathway from the MOFC to the MPFC. Furthermore, the immediate condition also reduced the connectivity from the MOFC to the dlPFC, which indicated that competition between these regions occurred under the immediate condition.

### L-TD

**Fig. 5:**
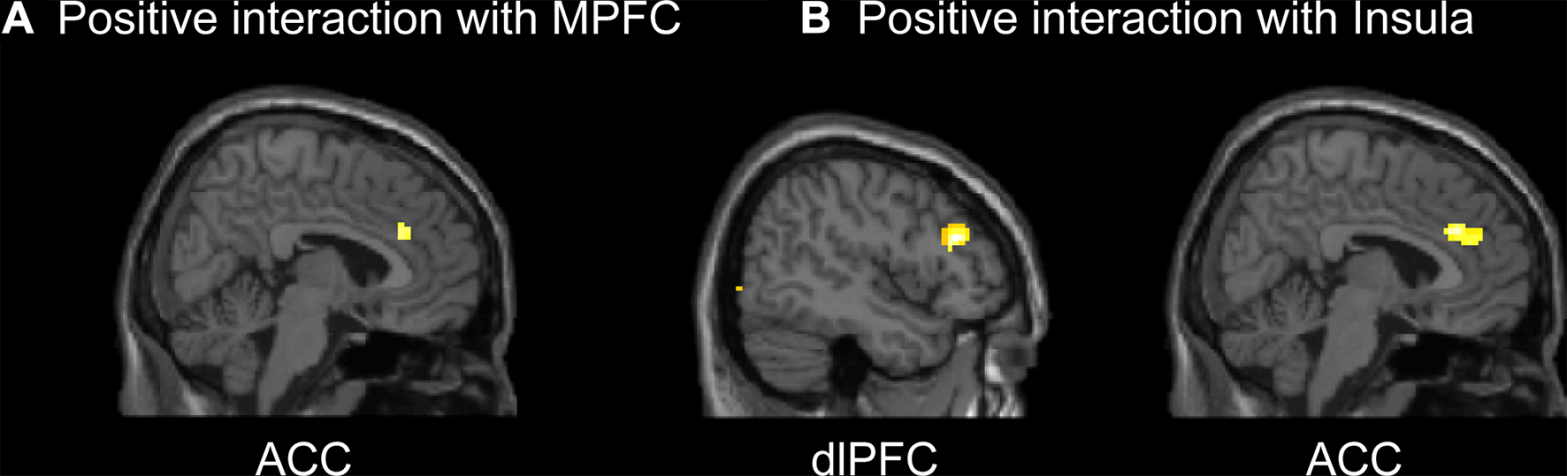
Psychophysiological interaction (PPI) results for all participants in the L-TD. **A** Regions that showed a significant interaction with activity in the medial prefrontal cortex (MPFC) during the immediate condition compared to the delayed condition. **B** Regions that showed a significant interaction with activity in the insula during the immediate condition compared to the delayed condition. ACC, anterior cingulate cortex; dlPFC, dorsolateral prefrontal cortex.

In our previously published findings, decision-related activation was found in the frontal-parietal network (including the dlPFC, lateral orbitofrontal cortex, and PPC), thalamus and right striatum across all decision epochs of losses [17]. We also found that the ACC, insula, MPFC, superior frontal gyrus, and PCC showed significantly enhanced activation when money was lost immediately. In the PPI analysis, we focused on the lateral prefrontal cortices and the MPFC-cingulate-insula network, including the MPFC, ACC and insula, because these regions have been implicated in aversive stimuli and negative emotional states [36, 37, 39, 50]. PPI analysis revealed greater functional coupling within the MPFC-cingulate-insula network and between the dlPFC and MPFC-cingulate-insula network upon comparing the immediate condition with the delayed condition (Fig. 5, Table S2). Specifically, the immediate condition enhanced the functional connectivity between the MPFC and ACC (*x* = 6, *y* = 30, *z* = 27, *t*-score = 3.23), the functional connectivity between the right insula and ACC (*x* = 3, *y* = 33, *z* = 27, *t*-score = 4.54), and between the right insula and right dlPFC (*x* = 48, *y* = 30, *z* = 24, *t*-score = 5.35).

**Fig. 6:**
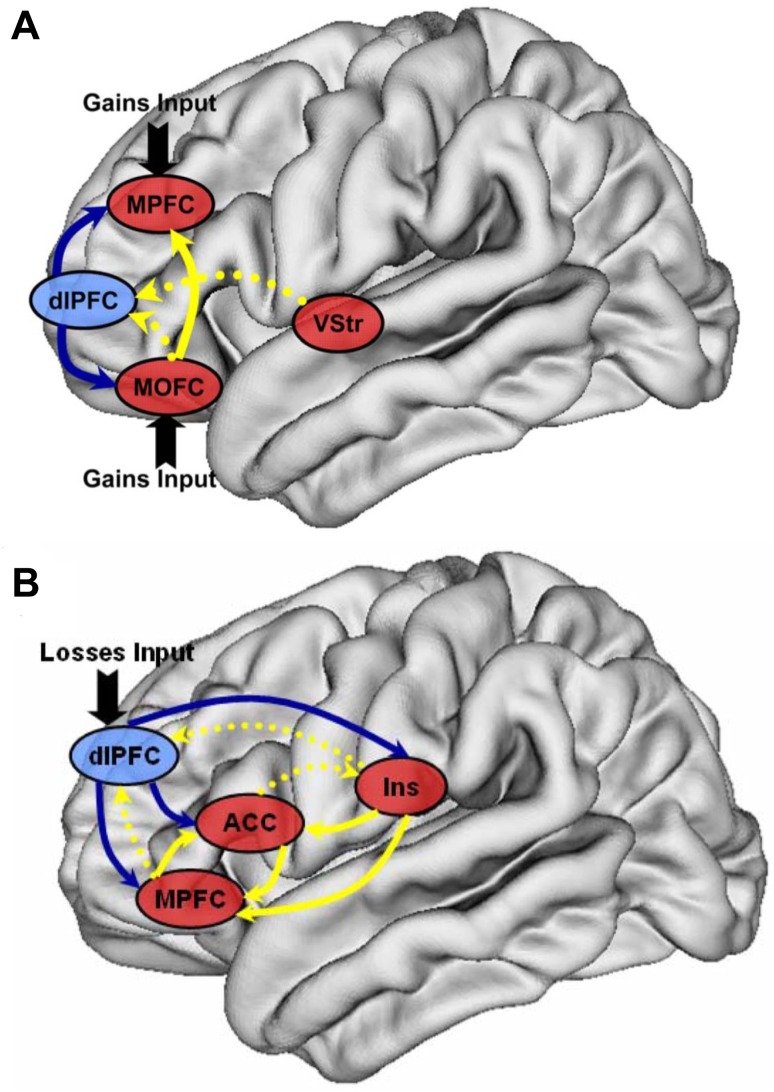
Schematic of the optimal dynamic causal model. **A** The optimal DCM in the G-TD. The main effect of the experimental manipulation (immediate condition) enhanced the connectivity from the dlPFC to the MOFC and MPFC as well as the connectivity from the ventral striatum to the dlPFC. The modulating effect of the immediate condition on the connectivity from the MOFC to the MPFC and dlPFC was negative. **B** The optimal DCM in the L-TD. The immediate condition enhanced the connectivity from the dlPFC to the MPFC, ACC, and insula as well as the bidirectional connectivity between the MPFC and ACC. The immediate condition also increased the connectivity from the insula to the MPFC and ACC. Connectivity from the insula and MPFC to the dlPFC increased during the immediate condition, as did that of the ACC to the insula. Solid lines for *P* < 0.05, corrected; dashed lines for *P* < 0.05, uncorrected. Blue lines indicate effective connectivity that began in the dlPFC; yellow lines indicate effective connectivity that began in the medial-striatal regions (**A**) or medial-cingulate-insula regions (**B**).

The DCM analysis revealed a preference for model 3 in which all the choices (regardless of delay) posited a significant influence on the bilateral dlPFC (Figs. 4B and 6; also see Fig. S4). In this optimal model, the immediate condition enhanced the connectivity from the bilateral dlPFC to the MPFC-cingulate-insula network (Fig. 4B). The immediate condition also enhanced local connectivity within the MPFC-cingulate-insula network, with stronger connectivity from insula to MPFC and ACC, and bidirectional connectivity between MPFC and ACC.

## Discussion

The aim of this study was to investigate whether dynamic interactions within intertemporal decision-related brain regions were different between gains and losses domains, and whether they were modulated by the immediate condition in which money was available immediately. Our previous studies identified multiple regions involved in temporal discounting and preliminarily established whether the underlying functional interactions between these regions showed differences between the gains and losses domains [17, 18]. The present study revealed two main findings. First, we found that gains were specifically evaluated in the medial regions, including the MPFC and MOFC, whereas losses were evaluated in the lateral regions (the dlPFC). Second, the immediate condition consistently modulated the functional coupling between the dlPFC and the medial-striatal regions in the G-TD, and between the dlPFC and the MPFC-cingulate-insula regions in the L-TD.

### Where Do the Choice Stimuli Enter?

Our results from the DCM analysis revealed a systematic difference in the evaluation of intertemporal choices between the gains domain and the losses domain. This suggested that there might be distinct mechanisms underlying the evaluation of intertemporal choice in the domains of gains and losses.

Specifically, in the G-TD, the DCM analysis indicated that the choice stimuli input was connected to the medial frontal cortices, including the MPFC and MOFC, which evaluated the subjective values of all gains, regardless of delay. These results are consistent with a substantial body of research implying that the mesolimbic regions are involved in the processing of anticipated rewards [27, 51–53]. The MOFC is involved in coding the relative values of different reward stimuli [24, 54, 55] and in updating the incentive value of outcomes in response to devaluation [56]. Given the role of the MPFC in integrating various kinds of reward value information [16, 26, 35, 57, 58], the MPFC and MOFC might integrate the anticipated subjective utility of future gains.

In the L-TD, the DCM analysis indicated that the choice stimuli input was connected with the bilateral dlPFC which had been implicated in prospective processing and future planning [30, 59]. These results are consistent with the arguments that decision makers might experience “savor” from anticipating future gains when a positive outcome is delayed [60, 61] and that they might experience “dread” from anticipating future losses when the outcome is negative [7, 38, 50]. Our finding that all choice stimuli were connected to the dlPFC might suggest that individuals experienced a strong feeling of dread as a result of anticipating the subjective utility associated with losses. Together with our previous reports [17, 18], participants preferred more SS losses than SS gains, indicating that the fear of future losses was a greater motivation than the pleasure associated with future gains. Therefore, the dlPFC might exert greater cognitive control to compete with the strong emotional tendency to avoid larger losses.

At the same time, the difference of DCM results between the gains and losses domains, especially the differential role of the dlPFC, raised a question about the role of the dlPFC in gains and losses. There are several potential explanations. First, there might be multiple valence-dependent valuation signals and neural processes which evaluate the subjective utility of gains and losses. The finding that choice stimuli enter on different regions in G-TD and L-TD might indicate different valuation processing of positive and negative outcomes. Specifically, the medial regions might be more sensitive to gains, whereas the dlPFC may be particularly sensitive to losses. This potential explanation is consistent with previous studies which found that lateral areas, including the dlPFC, are significantly activated in negative utility processing [17, 55, 62]. Another potential explanation is that losing money is correlated with negative motivation. Although the findings that greater preference for SS losses than gains might suggest that losses involve negative emotions, this does not indicate that such patterns of intertemporal choice of losses are irrational. Individuals might need more cognitive control to override the tendency to delay the acquisition of smaller losses in order to avoid larger losses. Therefore, activation in the dlPFC might suggest such an attempt to override the prepotent aversion to delay upcoming losses [48].

### How Do Brain Regions Interact to Arrive at a Decision?

Our findings might clarify several issues in the brain networks of intertemporal choice.

First, in the G-TD, our findings revealed a “two-system” involvement in the brain networks of intertemporal choice: the mesolimbic regions (*i.e*., the MPFC and MOFC) and the lateral cortical regions (*i.e*., the dlPFC). In contrast to McClure *et al.*’s [14, 15] notion that delayed and immediate rewards are separated, our DCM analysis of gains indicated that the mesolimbic regions respond to both delayed and immediate rewards by showing that all choice stimuli inputs were connected to the MPFC and MOFC. The dlPFC, in our findings, modulated neural responses to rewards, indicating its role in cognitive control of the delay discounting of gains. This finding is consistent with a recent study conducted by Hare *et al.* [35], which found that the medial frontal regions evaluate the common valuation signal, whereas the dlPFC plays a critical role in self-control by modulating the value signal encoded in the medial frontal regions. Thus, in the context of gains, the dlPFC might be involved in inhibiting the tendency to choose the SS rewards [13].

Second, our results might indicate distinct roles of the MOFC and MPFC in receiving a common value signal. This result is consistent with a recent study which reported dissociable roles of the MOFC and MPFC in the rat [63], suggesting that the MOFC plays a direct role during decision-making that extends simple outcome monitoring and representation, while the MPFC is mainly associated with representing rewards. Specifically, we found that the reciprocal connections from the MOFC to the dlPFC and the MPFC were both negative in G-TD, indicating an inhibitory effect of MOFC in intertemporal choice. Several studies have indicated that the MOFC might be involved in updating the incentive value of outcomes in humans [56] and that the MOFC might even be the critical region that mediates impulsive choice in animals [64]. Still, lesion studies have reported contradictory results on the directional roles of the MOFC in impulsive choices: either a decrease after the development of lesions in the MOFC [56, 65, 66] or an increase in impulsive choices [64, 67]. Our results suggest a unidirectional inhibitory role of the MOFC in impulsive choices. Together with the above research, our results indicate that the functional interactions between the MOFC and the MPFC convey motivational incentives based on the subjective value of gains by regulating the differential engagement of the MPFC, while the functional interactions from the MOFC to the dlPFC convey impulsivity information for inhibiting behavior.

Third, our findings in the L-TD revealed that intertemporal choices of losses recruited the integration of the neural circuitry of negative emotion-related regions and the lateral prefrontal cortex. This notion is supported by our findings that the immediate condition enhanced the forward connectivity from the bilateral dlPFC to the MPFC-cingulate-insula network and the connectivity within the MPFC-cingulate-insula network. Compared with gains, losses provoke emotional responses associated with fear or dread [24]. Neuroimaging studies have shown that the insula is activated in response to the negative emotions induced by social exclusion [68], unfairness [36], a disgusting odor [69], or the feeling of dread induced by a delayed unpleasant outcome [50]. Other studies have shown that the ACC responds to a variety of negative utilities or expected values which are derived from the consumption of goods and services [50, 70–72]. We also found that the immediate condition enhanced the functional interactions between the insula, ACC, and MPFC, which indicated that immediate losses might invoke stronger negative emotions. The forward connectivity from the dlPFC to these emotion-related regions was also enhanced in the immediate condition, suggesting that the participants attempted to override the prepotent aversion to delay the upcoming losses. Above all, our results indicate that the dlPFC plays a key role in biasing the emotion-related network.

## Limitations

It should be noted that our study has several inherent limitations from the original study [17]. The relatively small sample size of our study might bias the selection of the seed regions, which mainly depends on statistical thresholding in the whole-brain analysis. In the fMRI analysis, we did not include the age and gender as covariates to control their effects. In addition, the experimental design in which the gain and loss trials were separately presented in different blocks might cause a contextual effect. Future studies should improve the experimental design by presenting the gain and loss trials in a randomized way within the same block, or draw from paradigms that directly examine the processes involved in risky decision making [73].

## Conclusions

In summary, the results of this study provide evidence that separate neural networks underlie the intertemporal choice of gains and losses. We found two distinct valuation systems for gains and losses. In addition, compared with delayed choices, immediate options modulated functional integration between the lateral prefrontal cortex and mesolimbic regions in gains, and functional integration between the lateral prefrontal cortex and emotional regions in losses. These results indicate that valence might exert its influence *via* distinct mechanisms in the gain and loss domains. In particular choices, especially when one option is immediately available, the subjective values of the options might be modulated by different regions between gains and losses. Above all, the separate neural networks for gains and losses enrich our understanding of the neural mechanisms in intertemporal choice.

## Electronic supplementary material

Below is the link to the electronic supplementary material.

Supplementary material 1 (PDF 519 kb)
