# Supplementary material for: Separate Neural Networks for Gains and Losses in Intertemporal Choice
Source: Neurosci Bull. 2018 Aug 7;34(5):725–35. doi: 10.1007/s12264-018-0267-x (PMC6129240; doi:10.1007/s12264-018-0267-x)
Supplement: Supplementary file 1 — Supplementary material 1 (PDF 519 kb) [file 12264_2018_267_MOESM1_ESM.pdf]

Supplementary Materials

Separate Neural Networks for Gains and Losses in Intertemporal Choice

Yang-Yang Zhang[1, #], Lijuan Xu[2,3, #], Zhu-Yuan Liang[4,5, #], Kun Wang[2], Bing Hou[2,3], Yuan Zhou[4,5], Shu Li[4,5,6,*], Tianzi Jiang[2,3,7,8,*]

[1]School of Psychology, Shaanxi Normal University, Xi’an 710062, China

[2]Brainnetome Center, Institute of Automation, University of Chinese Academy of Sciences, Beijing 100190, China

[3]National Laboratory of Pattern Recognition, Institute of Automation, University of Chinese Academy of Sciences, Beijing 100190, China

[4]CAS Key Laboratory of Behavioral Science, Institute of Psychology, Beijing 100101, China

[5]Department of Psychology, University of Chinese Academy of Sciences, Beijing 100049, China

[6]Department of Psychology and Behavioral Sciences, Zhejiang University, Hangzhou 310028, China

[7]CAS Center for Excellence in Brain Science and Intelligence Technology, Chinese Academy of Sciences, Beijing 100190, China

[8]The Clinical Hospital of Chengdu Brain Science Institute, MOE Key Lab for Neuroinformation, University of Electronic Science and Technology of China, Chengdu 610054, China

[#]Yang-Yang Zhang, Lijuan Xu and Zhu-Yuan Liang have contributed equally to this work.

Table S1. Results from psychophysiological interaction analysis in the G-TD for the contrast of the immediate condition (choices that involve immediate options) *versus* the delayed condition (choices that involve only delayed options).

Interactions	MNI coordinates			t-value	BA	Direction of Connectivity
	x	y	z			
Connectivity with Medial Orbitofrontal Cortex						
Ventral Striatum	-6	6	-15	3.25		Positive
L Dorsolateral Prefrontal Cortex	-48	45	3	3.53	46,10	Positive
Connectivity with Medial Prefrontal Cortex						
R Dorsolateral Prefrontal Cortex	45	42	30	3.51	46,10	Positive
Connectivity with Ventral Striatum						
Medial Orbitofrontal Cortex	15	60	-6	3.71	10,11	Positive
Medial Prefrontal Cortex	3	60	12	4.12*	10	Positive
R Dorsolateral Prefrontal Cortex	45	39	30	4.23*	46,10	Positive

Coordinates of the peak voxel are reported in MNI space. $*P < 0.05$ corrected for multiple comparisons at the combined threshold of $P < 0.01$ and a cluster size of at least 74 resampled voxels determined by a Monte Carlo simulation using the AFNI AlphaSim program. For completeness, peaks are reported for all clusters ≥5 voxels at $P < 0.005$ uncorrected. **R**, right; **L**, left; **BA**, Brodmann's area; (x, y, z), coordinates of the primary peak locations in the Montreal Neurological Institute (MNI) space.

Table S2. Results from psychophysiological interaction analysis in the L-TD for the contrast of the immediate condition (choices that involve immediate options) *versus* the delayed condition (choices that involve only delayed options).

Interactions	MNI coordinates			t-value	BA	Direction of Connectivity
	x	y	z			
Connectivity with Medial Prefrontal Cortex						
Anterior Cingulate Cortex	6	30	27	3.23	32	Positive
Connectivity with R Insula						
Anterior Cingulate Cortex	3	33	27	4.54*	32	Positive
R Dorsolateral Prefrontal Cortex	48	30	24	5.35*	46,9	Positive

Coordinates of the peak voxel are reported in MNI space. *$P < 0.05$ corrected for multiple comparisons at the combined threshold of $P < 0.01$ and a cluster size of at least 74 resampled voxels determined by a Monte Carlo simulation using the AFNI AlphaSim program. For completeness, peaks are reported for all clusters ≥5 voxels at $P < 0.005$ uncorrected. **R**, right; **L**, left; **BA**, Brodmann's area; (x, y, z), coordinates of the primary peak locations in the Montreal Neurological Institute (MNI) space.

Table S3. Coordinates of the seed regions in the G-TD in the DCM analysis.

Brain Regions	Abbreviations	BA	Coordinates		
			x	y	z
Medial Prefrontal Cortex	MPFC	10	6	57	9
Medial Orbitofrontal Cortex	MOFC	11	3	48	-15
Ventral Striatum	VStr		-6	9	-6
R Dorsolateral Prefrontal Cortex	R.dlPFC	46,10	45	39	30
L Dorsolateral Prefrontal Cortex	L.dlPFC	46,10	-48	45	3

R, right; **L**, left; **BA**, Brodmann's area; (x, y, z), coordinates of the primary peak locations in the Montreal Neurological Institute (MNI) space.

Table S4. Coordinates of the seed regions in the L-TD in the DCM analysis.

Brain Regions	Abbreviations	BA	Coordinates		
			x	y	z
Medial Prefrontal Cortex	MPFC	10/11	9	42	-6
Anterior Cingulate Cortex	ACC	24/32	3	36	15
Insula	Insula	13	42	-15	9
R Dorsolateral Prefrontal Cortex	R.dlPFC	46,9	48	30	24
L Dorsolateral Prefrontal Cortex	L.dlPFC	46,9	-48	30	24

R, right; **L**, left; **BA**, Brodmann's area; (x, y, z), coordinates of the primary peak locations in the Montreal Neurological Institute (MNI) space.

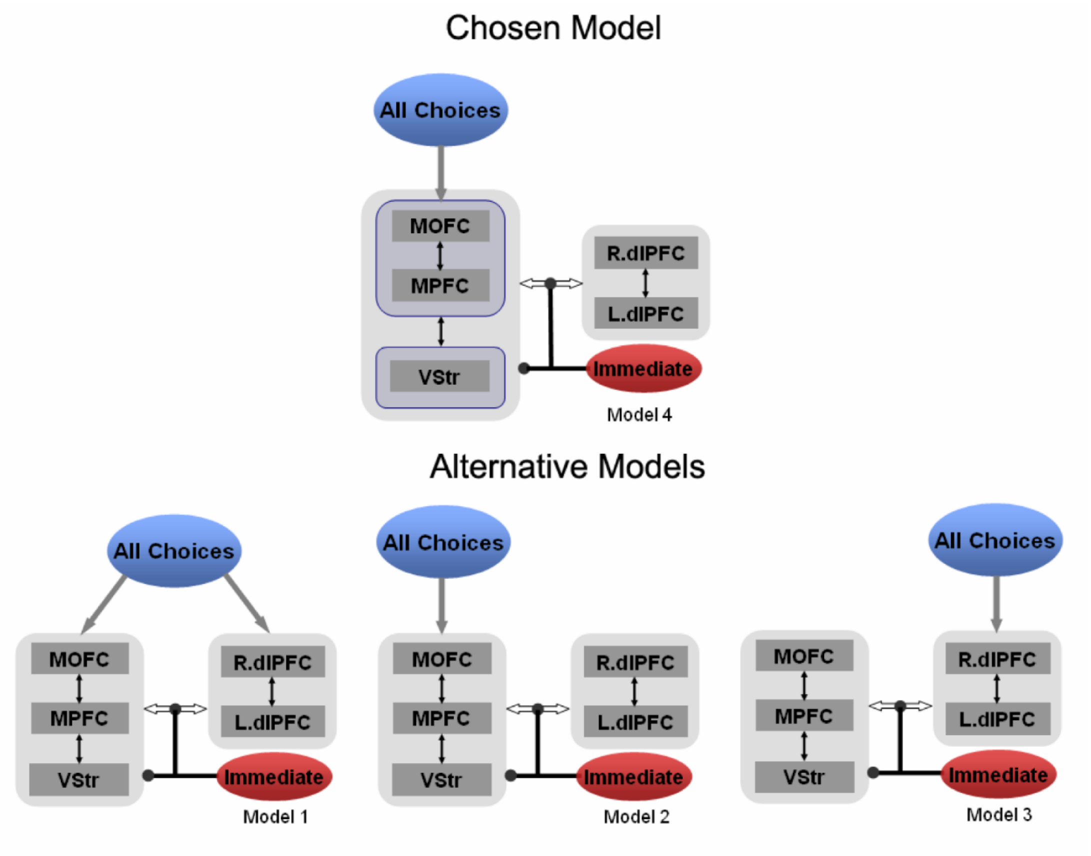

Fig. S1 Models tested using the DCM for the G-TD. Different DCM models that included the medial prefrontal cortex (MPFC), medial orbitofrontal cortex (MOFC), ventral striatum (VStr), and bilateral dorsolateral prefrontal cortex (dlPFC) as regions of interest (ROIs) were examined. The four models differ with respect to where the driving inputs (all choices, regardless of delay) were injected: in all the five regions (model 1); only in the medial-striatal regions, including the MPFC, MOFC, and VStr (model 2); only in the bilateral dlPFC (model 3); and only in the MPFC and MOFC (model 4). All the models assumed bidirectional intrinsic connections between these five regions. The interactions within the medial-striatal network and between the dlPFC and medial-striatal network were modulated by the immediate condition. All models were estimated by means of DCM, and then evaluated using Bayesian model selection. DCM: Dynamic Causal Modeling.

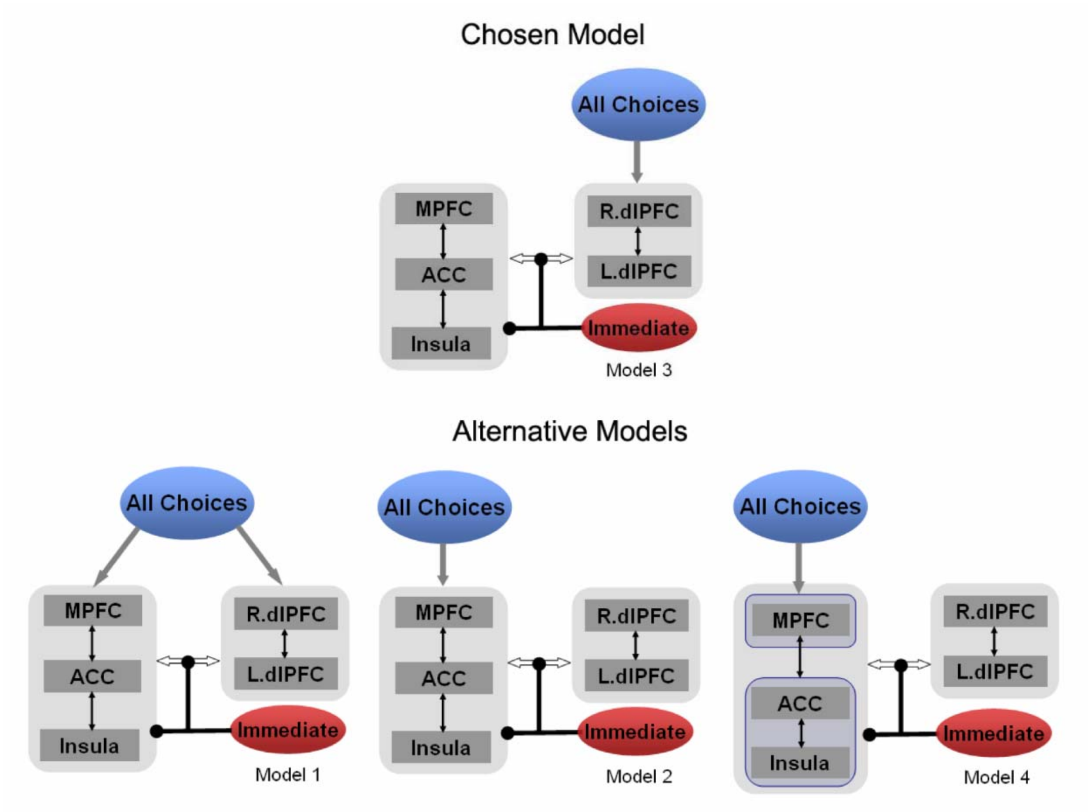

Fig. S2 Models tested using DCM for the L-TD. Different DCM models included the medial prefrontal cortex (MPFC), anterior cingulate cortex (ACC), insula, and bilateral dorsolateral prefrontal cortex (dlPFC) as regions of interest (ROIs). The four models differ with respect to where the driving inputs (all choices, regardless of delay) were injected: in all the five regions (model 1); only in the medial-cingulate-insula regions, including the MPFC, ACC, and insula (model 2); only in the bilateral dlPFC (model 3); and only in the MPFC (model 4). All the models assumed bidirectional intrinsic connections between these five regions. The interactions between the dlPFC and medial-cingulate-insula network and the connections within the medial-cingulate-insula network were modulated by the immediate condition. All models were estimated by means of DCM, and then evaluated using Bayesian model selection. DCM: Dynamic Causal Modeling.

(A) Fixed-effect Bayesian model selection

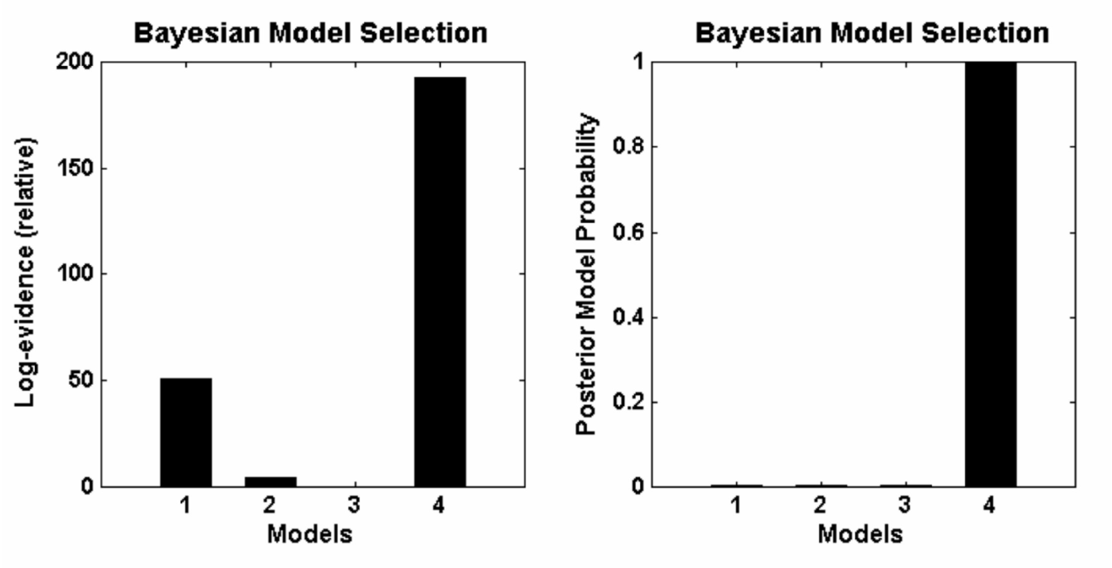

(B) Random-effect Bayesian model selection

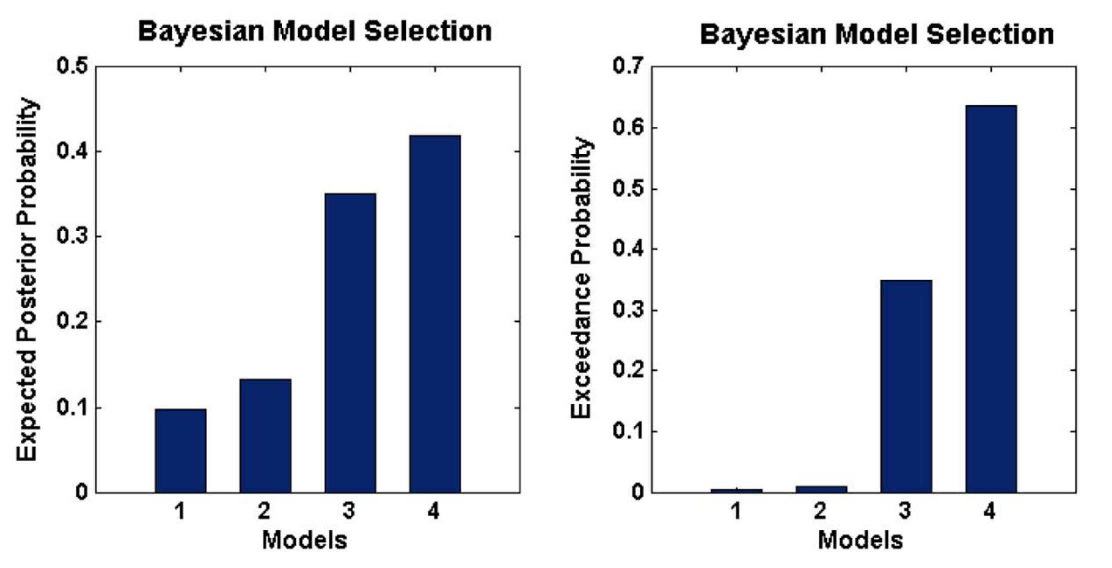

(C) This additional analysis was used to examine the difference between the chosen model (model 4) and the second-best model (model 3). Using random-effect Bayesian model selection, we found that there was an 85% *versus* 15% probability that model 4 was the more likely model compared with model 3. Posterior distribution and exceedance probability for the two models are presented.

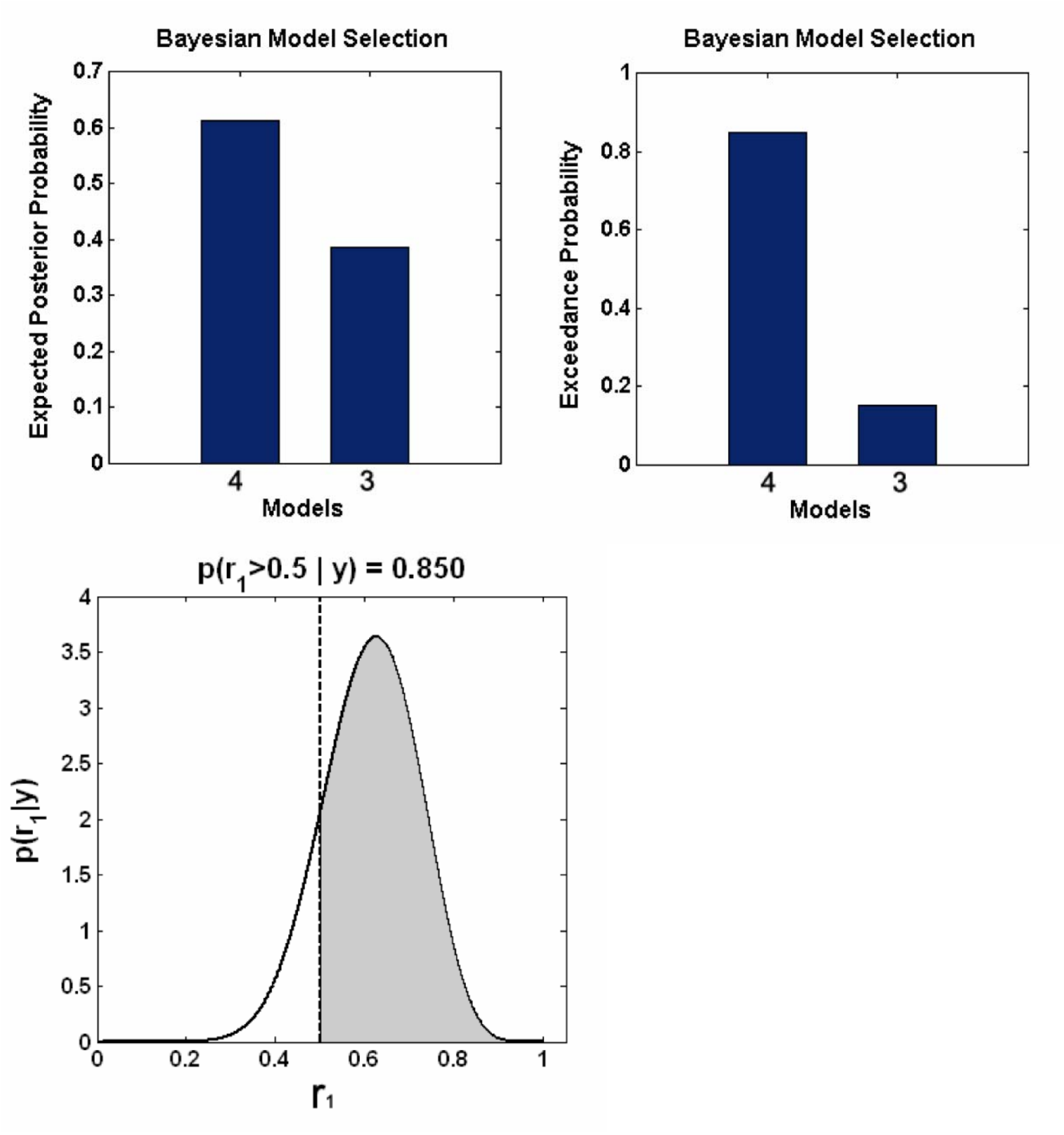

Fig. S3 Comparison of the four DCM models shown in Figure S1. We compared the four models using Bayesian model selection. The selection procedures estimated the posterior model probability of each model given the data. The fixed-effect (**A**) and random-effect (**B**) Bayesian model selection both showed strong evidence in favor of model 4. (**C**) The random-effect Bayesian model selection analysis between model 4 and model 3 which allows to quantify the probabilities that a given model is more likely than any other model tested.

(A) Fixed-effect Bayesian model selection

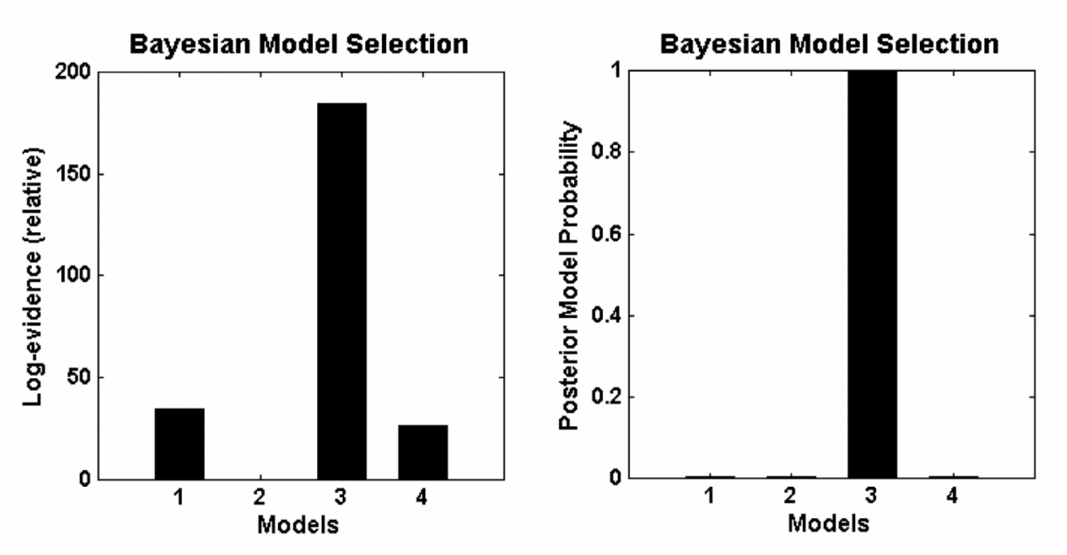

(B) Random-effect Bayesian model selection

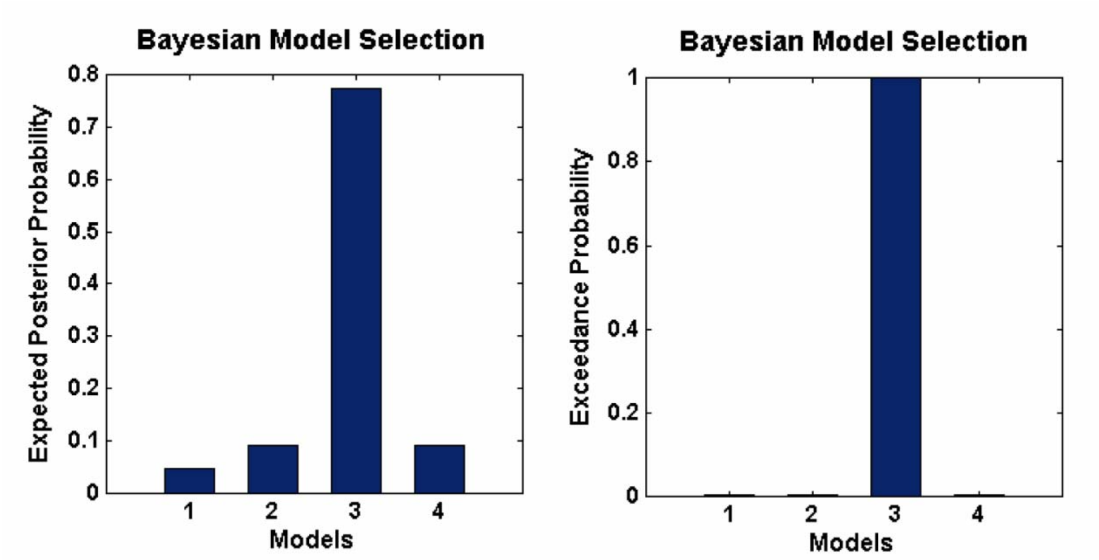

Fig. S4 Comparison of the four DCM models shown in Figure S2. We compared the four models using Bayesian model selection. The selection procedures estimate the probability of each model given the data. The fixed-effect (**A**) and random-effect (**B**) Bayesian model selection both showed strong evidence in favor of model 3.
